# Continuous flow photocatalytic reactor for degradation of selected pollutants: Modeling, kinetics, mineralization rate, and toxicity assessment

**DOI:** 10.1016/j.heliyon.2024.e40019

**Published:** 2024-10-31

**Authors:** Qasim Jamil, Boštjan Žener, Ula Putar, Lev Matoh

**Affiliations:** Faculty of Chemistry and Chemical Technology, University of Ljubljana, Večna Pot 113, 1000, Ljubljana, Slovenia

**Keywords:** Advance oxidation processes, Photoreactor, Emerging contaminants, Pollutant remediation, Operating parameters

## Abstract

This study focused on developing and evaluating a continuous flow photoreactor with an immobilized photocatalyst. The titanium dioxide powder was deposited on glass beads and packed into sequentially connected columns surrounded by LED lamps. The volume of the reactor without beads is 2.4 L, and with beads, 0.8 L. The photocatalytic efficiency of the reactor was evaluated by observing the degradation of Plasmocorinth B pollutant and selected pharmaceuticals (ibuprofen, sulfamethoxazole and diclofenac) at different flow rates under illumination of varying number of lights in deionized water and ISO medium. CFD simulations were performed to analyze the velocity and radiation field. The relationship between mass transfer and reaction kinetics was quantitatively evaluated by calculating the Peclet number, Damköhler number, and mass transfer coefficients. Total organic carbon (TOC) was also measured in the resulting solutions to determine the rate of mineralization. The toxicity tests were performed by exposing the solutions to the planktonic crustacean Daphnia magna for 48 h. The results showed that the number of lights directly and the flow rate inversely affected the degradation of the parent compound. At lower flow rates, total degradation of 87–97 % of the contaminants was observed in one flow and halving the light intensity resulted in a 10–15 % decrease in overall degradation. The toxicity tests showed that toxic transformation products were formed and were present until the complete degradation of the parent compound, after which they were also degraded. This study shows that the continuous flow photoreactor presents a potential solution for large-scale wastewater treatment.

## 1 Introduction

In recent decades, pollution from recalcitrant organic pollutants in wastewater has increased significantly and has become one of the leading causes of inadequate access to clean water. Even at trace concentrations (in μg.L^−1^), their persistence poses a potential environmental hazard to human health and aquatic life [[1], [2], [3]]. Moreover, climate change induced droughts, population growth, and rapid industrialization are expected to elevate the demand for potable water by almost 50 % by 2050 [4]. Water reclamation is an appealing option among the solutions to mitigate this problem. Therefore, extensive work has been carried out on treating organic pollutants dissolved in water, such as textile dyes, pharmaceuticals, endocrine disruptors, etc. [[5], [6], [7], [8], [9]]. To date, various conventional treatment technologies, such as biological, chemical, and physical, have been developed for their removal from wastewater. However, they are ineffective as they result in only partial removal or separation and the creation of more toxic residues [[10], [11], [12], [13]]. Thus, an efficient destructive method is required to completely mineralize these organic compounds.

Advanced oxidation processes (AOPs) have been extensively studied to treat water containing organic contaminants, as they can completely mineralize these persistent compounds [11,14,15]. Among other AOPs, semiconductor photocatalysis has proven to be an efficient approach to degrade the aforementioned pollutants completely. After absorbing photons with energy greater than its band gap, the photocatalyst produces highly reactive hydroxyl radicals (OH^.^) that attack non-selectively and, as a result, degrade very persistent organic compounds to yield H_2_O, CO_2_ and inorganic ions [[16], [17], [18]]. Other advantages of photocatalysis are that it works under ambient conditions and has lower operating costs as no additional chemicals are required. This makes it an energy-efficient, chemical-free, and sustainable method [11,19,20]. Titanium dioxide (TiO_2_) as a photocatalyst has received a lot of attention owing to its advantageous properties, such as high efficiency, ease of synthesis, biocompatibility, and low cost. To exploit the phenomenon of photocatalysis for large-scale water treatment, photoreactors are being developed.

Photocatalytic reactors have been developed for various types of applications, including water treatment, hydrogen generation and air purification. These developed photoreactors can be classified into different types based on their design features, such as geometry (tubular and cylindrical), light source (natural or artificial, lamps or LED), size (pilot scale or microreactors) and photocatalyst loading (suspended or immobilized) [19,21,22]. The light source plays a crucial role in the reactor, and artificial light sources such as LEDs are advantageous due to their stability, energy efficiency, and concentrated wavelength [23,24]. Based on photocatalyst loading, the most common configurations are suspended and immobilized, where photoreactors with suspended photocatalysts require additional recovery processes that can be costly and inefficient. In general, the pollutant degradation rate by suspended photocatalysts is higher than immobilized photocatalysts but the inefficiency in filtration poses a serious threat to the aquatic ecosystem [25,26]. These additional processes can be avoided by depositing the photocatalyst on a suitable support material such as glass, cellulose, stainless steel sheets, ceramics, silica, and polymer films [[27], [28], [29], [30]]. These support materials are also known as substrates and are selected based on their shape, which provides a larger surface area and ensures that light reaches the photocatalyst surface uniformly [31]. Moreover, a continuous flow reactor with immobilized photocatalysts can be easily developed, which is a more practical and feasible approach for the treatment of real wastewater.

The photoreactor design is crucial as several operating parameters such as types of light and its intensity, flow rate, type and concentration of contaminant, effective mass transfer, reactor volume, and photocatalytic material and dosage influence the overall performance of the reactor [32]. Although a lot of work has already been carried out on the development of photoreactors for water treatment, most of the research has focused on lab-scale photocatalytic reactors [33]. This lack of knowledge is the main limitation on its large-scale applicability. Therefore, a photoreactor with an effective design configuration needs to be developed to fill the gap between theoretical scientific knowledge and practical applications [10,11,19,34].

Photocatalytic degradation of pollutants does not necessarily lead to their complete mineralization as transformation products can also form during the degradation, which can be more toxic than the primary compound [35]. Therefore, in addition to measuring the degradation of primary compound, ecotoxicological study and total organic carbon (TOC) measurement is also required to properly evaluate the efficiency of the reactor. The ecotoxicological study is performed by exposing the primary compound and its degradation products to living organisms such as *Vibrio fischeri*, *Daphnia magna,* and *Lemna minor* etc [[36], [37], [38]].

In the present study, a pilot-scale, tubular and continuous flow reactor was set up with immobilized photocatalyst TiO_2_ on glass beads [20,39]. TiO_2_ was deposited on beads using the Sol-gel/TiO_2_ procedure described in Ref. [20]. It was immobilized using the dip-coating method. The effect of operating parameters such as light and flow rate was investigated by observing the degradation of the Plasmocorinth B dye solution. Numerical simulations were performed to analyze the flow and radiation field in the reactor. Moreover, kinetics and mass transfer analysis were also performed. Different pharmaceuticals were also degraded to evaluate its efficiency for variety of pollutants. The degree of mineralization was evaluated by measuring TOC and toxicity assessment was also conducted using *Daphnia magna*.

## 2 Materials and methods

### 2.1 Materials and chemicals

Chemicals were used as purchased: commercially available Aeroxide TiO_2_ P25 from Evonik; titanium (IV) butoxide (>97 %) from Sigma Aldrich; absolute ethanol (p.a.) and methanol (p.a.) from Honeywell; acetonitrile (ACN) (>99.9 %) from Sigma Aldrich; HCl (37 %) from Fluka; acetic acid (>99.8 %) from Sigma Aldrich; ammonia solution (puriss. p.a.) from Fluka/Honeywell; KH_2_PO_4_ (>99 %) from Fluka; polyethylene glycol/raffinose tuning solution from Shimadzu; Triton X-114 from Sigma Aldrich; plasmocorinth B (60 %) from Sigma Aldrich; ibuprofen, diclofenac and sulfamethoxazole from Fluorochem. The Milli-Q water filtration system was used to obtain ultrapure water (>18.0 MΩ cm^−1^ at 25 °C).

### 2.2 Reactor configuration

**Fig. 1:**
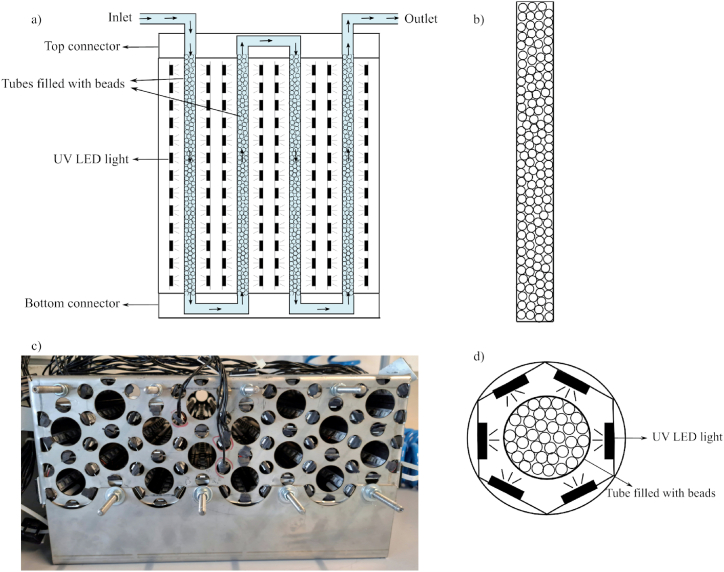
(a) Schematic diagram of front view of developed reactor, (b) schematic diagram of a column filled with coated glass beads, (c) top view of actual reactor without top connector, and (d) inside view of one place where column is inserted.

A tubular continuous flow reactor was developed, as shown in Fig. 1a. A suspension of P25 in TiO_2_ sol was prepared to immobilize the photocatalyst TiO_2_ on glass beads using the method developed by Matoh et al. [20]. The TiO_2_ sol was prepared by dissolving 1.5 mL of titanium (IV) butoxide in the 10 mL of absolute ethanol with 270 μL of 2 M HCl. The mixture was stirred for 3 h to obtain stable sol. After that, 1 g of P25 and 250 μL of Triton X-114 were added. As the characterization of the deposited material is the same as of the mentioned study [20] it has not been provided here. The photocatalytic reactor was made of stainless steel and consisted of sixteen columns loaded with TiO_2_-coated glass beads (Fig. 1b and c) connected in series from the above and below. The columns are made of poly (methyl methacrylate) material and have an average 90 % transmittance in the studied UV region (λ = 365 nm), which is more than other materials [40]. The total volume of the photocatalytic reactor was 2.4 L, as it consisted of 16 columns, each with a volume of 150 mL. The inner diameter of each column is 1.86 cm and length is 49 cm. After filling in the beads, the actual volume of the reactor is about 0.8 L. The radiation sources were commercially available LEDs (wavelength λ = 365 nm), and each column is hexagonally surrounded by six LED strips (henceforth, each strip will be referred as 1 lamp) with the same light intensity to ensure uniform light distribution across the column (Fig. 1d). Reflective foils were also fitted to limit light loss. This setup was connected to a control panel, which allows to individually turn the lamps on and off, and a pump to control the flow rate of the model pollutant stream.

### 2.3 Procedure

#### 2.3.1 Photocatalytic experiments

The photocatalytic efficiency of the designed reactor was tested by degrading the Plasmocorinth B (PB) dye (concentration γ = 12 mg L^−1^) and selected well-known pharmaceuticals (ibuprofen (IBU) γ = 10 mg L^−1^, diclofenac (DCF) γ = 4 mg L^−1^, sulfamethoxazole (SMX) γ = 10 mg L^−1^) in deionized water and ISO medium. The ISO medium is a mixture of CaCl_2_.2H_2_O, MgSO_4_.7H_2_O, KCl, and NaHCO_3_. The concentration of DCF was chosen to be lower than IBU and SMX due to its limited solubility. The experiments were also performed in an ISO medium to evaluate toxicity and the effects of the ISO medium on the performance. The photocatalytic efficiency of the developed reactor was evaluated at different flow rates (50, 75, 100, and 200 mL min^−1^) and under different number of lights (1, 2, 3, 4, 5, and 6 lamps) by degrading PB dye. IBU was degraded at two different flow rates (50 and 100 mL min^−1^) and under irradiance of 3 and 6 lamps to determine its impact on pharmaceuticals’ degradation. Table 1 shows the estimated light intensity of the lamps.

**Table 1:** Estimated light intensity of each number of lamps.

No. of lamps	Light intensity (x 10^−4^ W cm^−2^)
1	35
2	70
3	105
4	140
5	175
6	210

In each experiment, a 5 L solution of the specified pollutants was prepared using deionized water and ISO medium. Before the initial experiment, the reactor system was set up and reused for subsequent experiments. Prior to each run, the reactor was thoroughly cleaned and rinsed with deionized water under UV light to degrade any residual compounds from previous experiments.

To eliminate the adsorption effect of the pollutants on the photocatalyst, 1.5 L of the pollutant solution was cycled through the reactor in the dark, allowing the photocatalyst surface to become saturated. The remaining solution was then reserved for degradation under UV irradiation.

Efficiency was assessed in a continuous flow-through mode. The time required for the freshly prepared pollutant solution to pass through the reactor once was calculated based on the reactor volume (0.8 L with beads) and the flow rate. At the lowest studied flow rate of 50 mL min^-1^, it took about 16 min for the solution to pass through the entire reactor.

During the photocatalysis phase, samples were collected every 5 min to measure the concentration of the parent compound and total organic carbon (TOC) in the treated solution. Once the concentration of the parent compound stabilized, the results from the last three samples were used to calculate efficiency. Additionally, samples were collected for ecotoxicity testing.

The penetration of light into the beads in the center of the column is crucial for efficient photocatalytic reactions. Therefore, the penetration of light into the central part of the column was investigated by visual inspection and numerical simulations. Numerical simulation of the radiation field are presented in section 2.3.2. For the visual inspection, an experiment was conducted with a dye solution as a pollutant using only one column to test whether the external UV radiation can reach the center of the column. For this purpose, all beads were visually inspected after adsorption and photocatalysis. It was found that all beads turned purple after adsorption of the dye, but after photocatalysis the color disappeared, and all beads returned to their original whitish color. This shows that light of sufficient intensity reaches the central beads to activate the deposited catalyst.

The concentration of the dye was measured with an Agilent Cary 60 UV–Vis spectrophotometer in the wavelength range of 400–700 nm by observing the absorbance spectra of the solution. The absorbance value at 550 nm was used to record the difference in the concentration of the dye. For pharmaceuticals, the concentration was determined using the Agilent 1260 Infinity II HPLC system. Prior to the measurements, the samples were centrifuged to remove potential particles.

#### 2.3.2 Flow and radiation field simulations

The flow was assumed to be incompressible, isothermal, Newtonian, non-reactive and turbulent with constant physical properties. The governing equation are as follows:

Continuity equation:

(1) $$\frac{\partial \rho }{\partial t}+\nabla \cdot \left(\rho v\right)=0\text{,}$$

Momentum conservation equation,

(2) $$\frac{\partial \left(\rho v\right)}{\partial t}+\nabla \cdot \left(\rho \mathrm{vv}\right)=-\nabla P-\nabla \cdot \left(\mu \nabla v\right)\text{,}$$

where ρ,v,P,t and μ stands for density, velocity, pressure, time and dynamic viscosity of the fluid, respectively.

Radiative transfer equation (RTE)

(3) $$\nabla \cdot \left(I_{\lambda }\left(\vec{r},\vec{\;s}\right)\;\vec{s}+\left(a_{\lambda }+\sigma_{s}\right)I_{\lambda }\;\left(\vec{r},\vec{\;s}\right)\right)=a_{\lambda }n^{2}I_{b\lambda }+\left(\frac{\sigma_{s}}{4\pi }\right)\;\int_{0}^{4\pi }I_{\lambda }\;\left(\vec{r},\vec{s^{\prime }}\right)\;\phi \;\left(\vec{s}\;.\;\vec{s^{\prime }}\right)\;d\Omega^{\prime }$$

Where $\vec{r}$ shows position vector, $\vec{s}$ direction vector, $I_{\lambda }$ radiation intensity, depends on position vectors ($\vec{r}$) and ($\vec{s}$), $\vec{s^{\prime }}$ scattering direction vector, ϕ phase function, $\Omega^{\prime }$ solid angle, $\sigma_{s}$ scattering coefficient, $a_{\lambda }$ absorption coefficient and n refractive index.

**Fig. 2:**
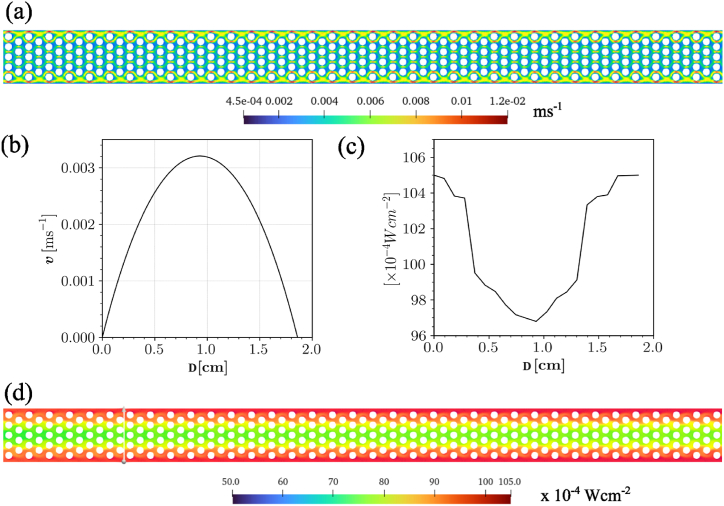
(a) Contour of velocity distribution in the photoreactor, (b) Poiseuille flow velocity profile at a cross-section near the tube's inlet, where D shows the diameter of tube, (c) incident radiation distribution at a cross-section near the tube's center and (d) contour of incident radiation mapping in the photoreactor.

The continuity (1), momentum (2) and radiation transfer equation (3) ensure mass, momentum and radiation conservation in the flow field. All the tubes inside the reactor are of the same size and shape therefore the numerical model of the reactor was simplified to one tube. One tube model is computationally less demanding and provides a better insight of the flow dynamics inside the reactor. The 2D CFD model was developed and analyzed in Ansys FLUENT 2023 R1, and the following boundary conditions were imposed: At the walls of the tube and spheres, no-slip velocity boundary condition (v=0) was applied. Velocity-inlet boundary condition is applied at the inlet of the tube, the velocity distribution at the inlet satisfies the wall shear boundary conditions and is described by the function $v_{x}=6.0v_{avg}\left(\frac{y}{D}\right)\left(1-\frac{y}{D}\right),v_{y}=0$, where $v_{avg}$ is the given velocity calculated from flow rate, and D is the diameter of the tube [41]. The velocity distribution at a cross-section near the tube's inlet is shown in Fig. 2. At the outlet, the pressure-outlet boundary conditions are specified. The fluid enters the tube at room temperature (T=300K). The walls of the channel are semi-transparent and irradiance of $105\;W.m^{-2}$ was provided at the top and bottom walls.

Fig. 2a and b shows the pattern of fluid flow through the reactor tube. The fluid passes over the beads, ensuring efficient transport of the pollutant solution through the coated photocatalyst. The walls of the beads decelerate the fluid increasing the contact time between the pollutant solution and the photocatalyst, resulting in enhanced degradation. However, the constant flow of solution through the inlet and outlet also maintains mass transfer.

Numerical investigations were also carried out to analyze the distribution of the radiation field within the reactor. Fig. 2c and d shows a higher light intensity closer to the walls, as there is no obstacle present there. The central beads are slightly less illuminated, but it rarely affects the efficiency of the reactor. The figure shows that more than 70 % radiation was able to penetrate the to central beads, which is sufficient to activate the photocatalyst.

#### 2.3.3 Total organic carbon (TOC) measurements

Each time while collecting the samples for the measurement of parent compound concentration, samples were also collected for the total organic carbon (TOC) measurement. The TOC in the solution was determined using Shimadzu TOC-L 5000A Analyzer with ASI-L feeder.

#### 2.3.4 Ecotoxicity tests

The detoxification efficiency of the developed photoreactor and the toxicity assessment of the transformation products formed during photocatalytic treatment were evaluated by comparing the toxicity of the untreated and photocatalytically treated pollutants solutions. The acute immobilization tests were performed according to OECD guidelines 202 [42] by exposing small planktonic crustacean *Daphnia magna* to the solutions for 48 h and afterwards their immobilization was checked. For every test, three replicates of ISO medium (control) and three replicates of each testing solution were prepared. Each replicate had 10 daphnids younger than 24 h that were exposed to 10 mL of ISO medium or tested solution. The mobility of the daphnids was visually observed after 24 and 48 h. The daphnid was considered immobile if it did not move for 15 s after gentle shaking of the test tray.

## 3 Results and discussions

### 3.1 Effect of flow rate

To ensure maximum photocatalytic degradation of pollutants in a continuous flow photoreactor, an optimal flow rate is required. The flow rate affects the residence time of the pollutant stream in the reactor and determines the contact time of the pollutants with the photocatalyst and the exposure to light. An appropriate residence time ensures maximum interaction between the photocatalyst and the pollutant, which accelerates photocatalytic degradation. This approach allows maximum degradation to be achieved up to a certain value, as a flow rate that is too low can lead to low mass transfer, which limits the interaction between the pollutant and the photocatalyst. To evaluate the efficiency at different flow rates, the PB dye solution was pumped through the reactor at a flow rate of 50, 75, 100 and 200 mL min^−1^ and the degradation was observed under the irradiation of 3 UV lamps.

**Fig. 3:**
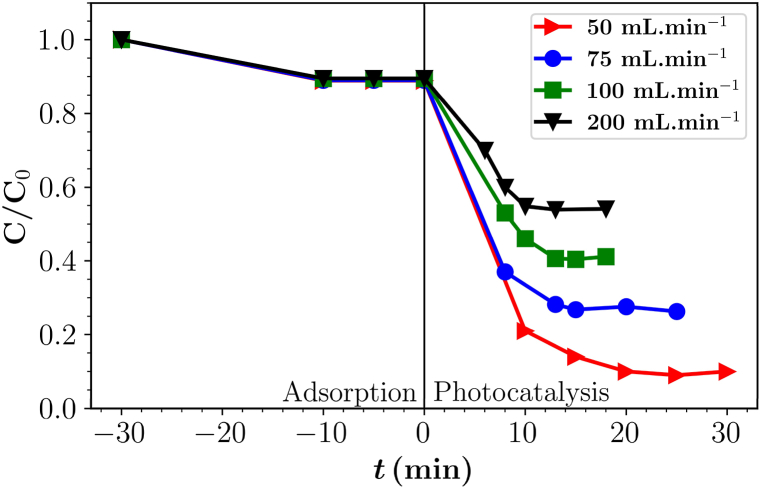
Photocatalytic degradation of parent compound PB at different flow rates with three lamps.

The overall degradation of the dye solution at different flow rates in one flow is shown in Fig. 3. The results show the degradation efficiency at flow rates of 50, 75, 100 and 200 mL min^−1^ were 88, 70, 60, and 54 % respectively which indicates the degradation efficiency is inversely proportional to the flow rate. These results are consistent with previous studies [43,44].

To investigate the effect of flow rate on the degradation of intermediates together with the parent compound, the TOC of the above experiment was measured. Table 2 shows the TOC concentration in the degraded PB dye solutions together with the percentage of TOC removal. These results are consistent with the results of the degradation of the initial compound, as the TOC removal of PB dye decreased from 26 to 1 % with increasing flow rate from 50 to 200 mL min^−1^. This decrease in TOC removal can be attributed to the shorter contact time of the contaminant with the photocatalyst at higher flow rates. The photocatalytic degradation of parent compound often leads to the formation of smaller compounds, also known as intermediates or transformation products, which still contribute to the TOC. Consequently, the efficiency of TOC removal is lower than that of the removal of the parent compound [45].

**Table 2:** Parent compound (PC) degradation and total organic carbon (TOC) removal of PB dye at different flow rates under the illumination of three lamps.

Flow rate (mL min^−1^)	PC degradation (%)	TOC removal (%)
50	89	26
75	70	11
100	60	2
200	55	1

### 3.2 Effect of number of lights

Light intensity also has an effect on the degradation process, as an increase in light intensity can lead to increased excitation of the photocatalyst and consequently increase the rate of degradation [14,46]. This increase is limited to a certain value, after which the degradation rate is independent of the light intensity [47], as the number of sites at which photons can trigger an oxidative reaction remain the same [48]. Moreover, increase in light intensity also leads to a waste of energy, which causes considerable costs [49]. Therefore, an optimal light intensity must be used to achieve a balance between maximum mineralization and minimum energy waste.

**Fig. 4:**
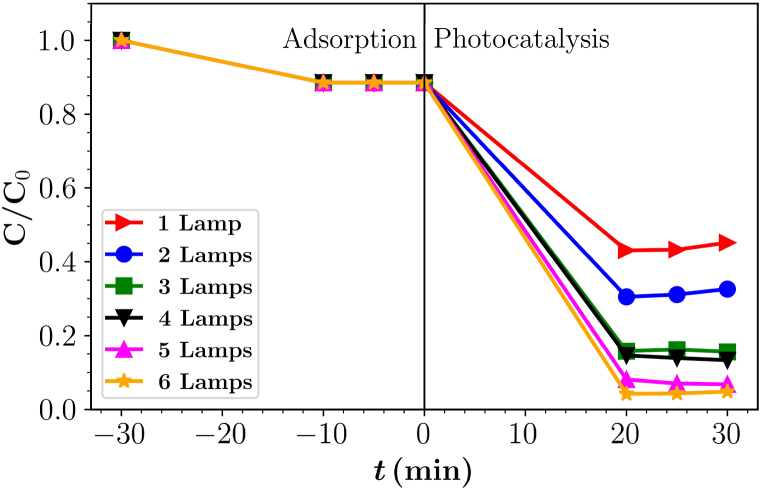
Photocatalytic degradation of parent compound PB under the illumination of different number of lamps at a flow rate of 50 mL min^−1^.

To determine the optimal number of lamps for the designed reactor, a series of experiments were performed at a flow rate of 50 mL min^−1^. The results show that the efficiency of degradation of PB dye parent compound under irradiation with 1, 2, 3, 4, 5 and 6 lamps is 55, 67, 88, 90, 93 and 95 %, respectively (Fig. 4). The increase in degradation efficiency is due to higher light intensity leading to a greater number of photons available to excite the activation sites, a greater likelihood of interaction between photons and the photocatalyst and more electron-hole pairs being formed [[50], [51], [52]].

**Fig. 5:**
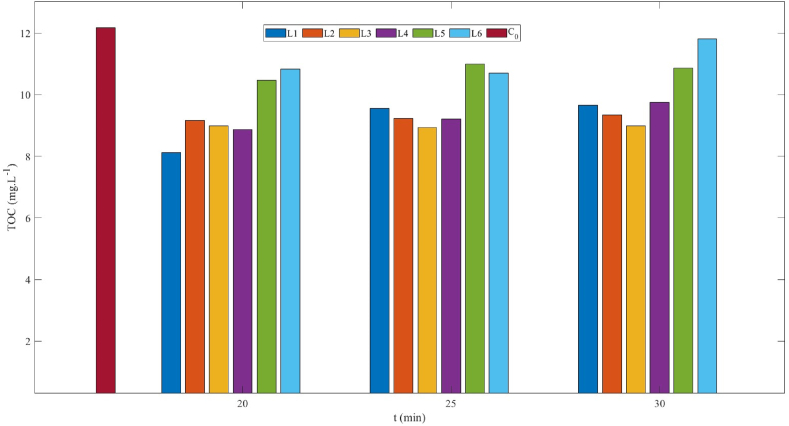
TOC concentration before (C_0_) and during PB degradation under different number of lamps after 20, 25 and 30 min of irradiation time at flow rate of 50 mL min^−1^.

TOC was also measured to investigate the effect of light intensity on the mineralization rate under the irradiance of a different number of LED lamps. The results of the TOC measurements are shown in Fig. 5 & Table 3 and show that the TOC removal efficiency after 30 min of irradiation is 21, 23, 26, 20, 11, and 3 % using 1, 2, 3, 4, 5, and 6 lamps respectively. TOC removal increased from 21 to 26 % when the number of lamps was increased from 1 to 3 and afterwards, it decreased from 20 to 3 % when the number of lamps was further increased from 4 to 6. The precise reasons for this discrepancy in results are still unknown and require further testing to clarify it.

**Table 3:** Parent compound (PC) degradation and total organic carbon (TOC) removal of PB dye under the illumination of different number of lamps at a flow rate of 50 mL min^−1^.

No. of Lights	PC degradation (%)	TOC removal (%)
1	55	21
2	67	23
3	89	26
4	90	20
5	93	11
6	97	3

In the experiments with a higher number of lamps, especially six lamps, the temperature of the outside part of reactor rose to 60 °C. This increase in temperature is because a significant portion of energy is lost as heat [49]. The temperature with three lamps was close to room temperature as less energy was lost as heat. Therefore, three lamps were chosen as the optimum light source and a cost-effective option.

### 3.3 Efficiency of pharmaceuticals’ degradation

To evaluate the performance of the designed reactor for the degradation of pharmaceuticals, degradation of three selected pharmaceuticals (IBU, SMX, DCF) was observed in deionized water and ISO medium. Initially, experiments were conducted to determine the optimum flow rate and light intensity by degrading IBU under different flow rates (50 and 100 mL min^−1^) and under the irradiance of different lamps (3 and 6) in the two mentioned media.

#### 3.3.1 Degradation of ibuprofen

##### 3.3.1.1 Effect of flow rate

**Fig. 6:**
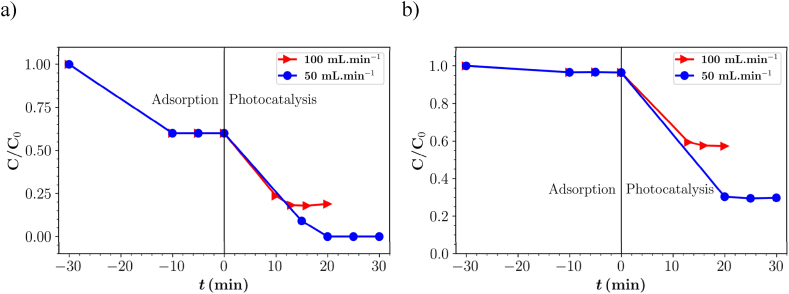
Photocatalytic degradation of IBU in (a) deionized water and (b) ISO medium at different flow rates with three lamps.

Fig. 6 shows the degradation efficiency of the parent compound IBU and TOC removal. The results indicated that the IBU degradation efficiency at a flow rate of 50 and 100 mL min^−1^ under 3 lamps in deionized water was 100 and 82 %, respectively. In ISO medium, the efficiency was 71 and 62 % at a flow rate of 50 and 100 mL min^−1^, respectively. Similar to the degradation of the PB dye at different flow rates, there is a decrease in degradation when the flow rate increases, as the contact time with the photocatalyst and the absorbed photons is shorter.

In the ISO medium, the efficiency decreases due to the presence of inorganic ions in the solution. Fig. 6b shows that the adsorption in the case of ISO medium was low compared to deionized water because of the adsorption of inorganic ions on to the photocatalyst surface impedes adsorption of pollutant and can also react with hydroxyl radicals, thus slowing down the photocatalytic degradation. Previous studies have also reported that inorganic ions negatively affect the photocatalytic degradation efficiency [[53], [54], [55]].

The TOC results (Table 4) of IBU degradation at different flow rates also showed a similar pattern observed in PB dye degradation. The efficiency of TOC removal was reduced from 76 to 46 % in deionized water and from 33 to 12 % in ISO medium when the flow rate was increased from 50 to 100 mL min^−1^.

**Table 4:** Parent compound (PC) degradation and total organic carbon (TOC) removal of IBU in deionized water and ISO medium at different flow rates under the illumination of three lamps.

Flow rate (mL.min^−1^)	Medium	PC Degradation (%)	TOC removal (%)
50	deionized water	100	76
100	deionized water	82	46
50	ISO medium	71	33
100	ISO medium	62	12

##### 3.3.1.2 Effect of number of lights

**Fig. 7:**
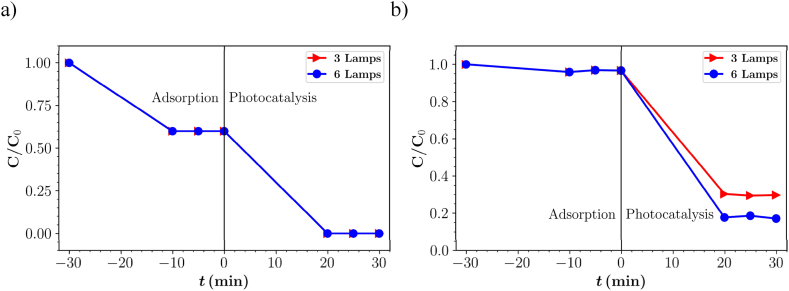
Photocatalytic degradation of IBU in (a) deionized water and (b) ISO medium with three and six lamps at flow rate of 50 mL min^−1^.

The degradation of IBU was observed using three and six lamps at a flow rate of 50 mL min^−1^ in deionized water and ISO medium. The results of degradation of the parent compound and TOC removal are shown in Fig. 7. In deionized water, IBU was completely degraded after 20 min of irradiation, while in ISO medium the efficiency of degradation with three and six lamps was 71 and 62 %, respectively. As explained in the previous section, the efficiency in the ISO medium was lower due to the presence of inorganic ions that hinder the adsorption of IBU on the active sites of the photocatalyst.

The TOC results (Table 5) showed that the removal efficiency was 33 % with three lamps and 20 % with six lamps in ISO medium. In deionized water, 76 % TOC removal was achieved with three lamps and 40 % with six lamps. Similar to the PB dye, the degradation of the parent compound of IBU increased with increasing number of lamps, while the TOC removal efficiency decreased.

**Table 5:** Parent compound (PC) degradation and total organic carbon (TOC) removal of IBU in deionized water and ISO medium under the illumination of three and six lamps at flow rate of 50 mL min^−1^.

Number of lamps	Medium	PC Degradation (%)	TOC removal (%)
3	Deionized Water	100	76
6	Deionized Water	100	40
3	ISO medium	71	33
6	ISO medium	62	20

#### 3.3.2 Sulfamethoxazole

**Fig. 8:**
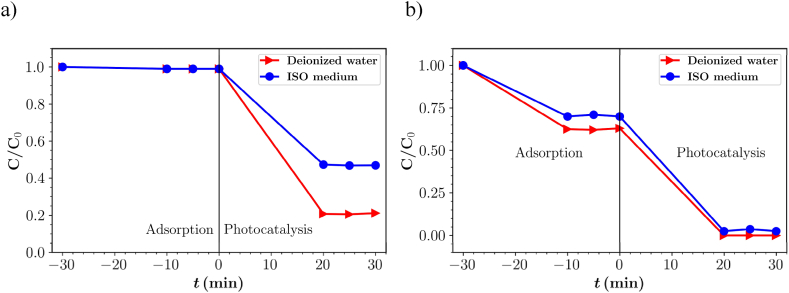
Photocatalytic degradation of a) SMX and b) DCF in different mediums with three lamps at flow rate of 50 mL min^−1^.

The degradation of SMX was observed under optimized conditions (flow rate of 50 mL min^−1^, irradiation with three lamps) in deionized water and ISO medium. The results of SMX degradation efficiency and TOC removal are shown in Fig. 8a. The results revealed SMX is only adsorbed to a very small extent on the surface of the photocatalyst in the dark due to the low electrostatic interactions between SMX (NH group) and the photocatalyst surface [56], whereas higher adsorption of IBU can be attributed to the functional group (-COOH), charge distribution and size of molecules [57]. This was also observed in the earlier study by Matoh et al. [20]. The degradation efficiency was 80 % in one flow-through mode in deionized water, while it decreased to 54 % in ISO medium under similar conditions. A similar trend was observed in the case of IBU in ISO, which can again be attributed to the presence of inorganic ions that have a negative effect on the photocatalytic degradation process of the pollutants.

The TOC results (Table 6) showed that the removal efficiency in deionized medium was 54 %, while in ISO medium it was 27 %. In the ISO medium, the low TOC removal is due to the low degradation of the parent compound and also intermediates.

**Table 6:** Parent compound (PC) degradation and total organic carbon (TOC) removal of SMX in deionized water and ISO medium at flow rate of 50 mL min^−1^ under the illumination of three lamps.

Flow rate (mL.min^−1^)	Medium	PC Degradation (%)	TOC Degradation (%)
50	Deionized Water	80	54
50	ISO medium	54	27

#### 3.3.3 Diclofenac

The photocatalytic performance of the designed reactor for the degradation of DCF in deionized medium and ISO medium was investigated under optimized conditions. Fig. 8b and Table 7 show the results of DCF degradation and TOC removal efficiency. The result showed that DCF was completely degraded in one flow through the reactor in deionized water and 97 % degraded in ISO medium. Due to the limited solubility of DCF, its concentration (4 mg L^−1^) was lower than the concentration of IBU and SMX (10 mg L^−1^).

**Table 7:** Parent compound (PC) degradation and total organic carbon (TOC) removal of DCF in deionized water and ISO medium at flow rate of 50 mL min^−1^ under the illumination of three lamps.

Flow rate (mL.min^−1^)	Medium	PC Degradation (%)	TOC Degradation (%)
50	Deionized Water	100	97
50	ISO medium	97	75

The TOC removal efficiency was 97 % in deionized water and 75 % in ISO medium. These results are consistent with the results of IBU and SMX, where the ISO medium reduced photocatalytic degradation.

### 3.4 Mass transfer and kinetic analysis

The reactors can possess various limiting regimes, based on the diffusion, advection and reaction time scale [58], such as (i) mass transfer limited regime dominates when the diffusion time is greater than the residence time and the reaction rate is greater than the diffusion time $\left(t_{diff}\gg t_{reaction}\right)$, (ii) kinetic limited regime takes place when reaction rate is lower than diffusion $\left(t_{reaction}\gg t_{diff}\right)$ and rate of reaction becomes the limiting factor and (iii) mass transfer and kinetics both limit the degradation process when the time scales of both are similar [59].

For in-depth understanding of the interplay between kinetics and mass transfer in the studied reactor system, (a) Peclet number, (b) Dam Köhler number (Da) and (c) mass transfer coefficients were calculated at the studied flow rates shown in Table 8. (a) The Peclet number (*Pe*) is a dimensionless number and represents the relative dominance of bulk flow convection compared to diffusion for mass transfer in the photoreactor. The equation used for calculations is $\text{Pe}=\frac{vL}{D}$, where v is the characteristic speed (m.s^−1^), L is the characteristic length (m) and D=μ/ρ is the diffusivity (m^2^.s^−1^) [60]. For the studied reactor system, moderate Peclet number was observed, and it ranges from 57 to 228 for the studied flow rates. These values show that convection is the main mode of mass transfer across all flow rates since *Pe* > 1. The increasing trend of *Pe* value was observed with an increasing flow rate. This indicates that increasing flow rate results in a more pronounced effect of convection compared to diffusion. It is also crucial to consider other parameters for proper evaluation of reactors, especially reaction kinetics. The degradation efficiency shows that at higher flow rate the degradation is lower, this suggests that flow rate plays a vital role in the reactor. Consequently, high *Pe* is leading to insufficient residence time for reaction.

**Table 8:** The calculated Peclet number, Damköhler number and mass transfer coefficients at the respective flow rates.

Flowrates (mL min^−1^)	Peclet number (*Pe*)	Damköhler number (Da)	Mass transfer coefficient (k_L_) (m s^−1^)
50	57.074	28	3.85 × 10^−5^
75	85.61	18	8.75 × 10^−6^
100	114.15	14	3.73 × 10^−6^
200	228.30	7	1.96 × 10^−6^

(b) For deeper understanding of the influence of flow rate, the Damköhler number was calculated. The Damköhler number (Da) is a dimensionless parameter that is used to understand the dynamics of reaction kinetics and mass transfer. It offers insightful information of the dominant regime controlling the overall degradation process. It is calculated as $\text{Da}=\tau kC^{n-1}$, where τ,k and C denote the residence time, reaction rate constant and initial concentration, respectively, while superscript n shows the order of the reaction which is 1. The reaction rate constant calculated was 0.0291 sec^−1^. The Da values range from 28 to 7 for studied flow rates. The observed trend of *Pe* aligns well with the calculated Da. The highest observed Da value of 28 at *Pe* = 57 shows that the reaction rate is faster compared to the residence time. Therefore, a more complete reaction occurred at flow rate of 50 mL min^−1^, which resulted in the highest degradation of 90 %. Further, the low Da values (18, 14, 7) at higher flow rates suggests that reaction rate might be becoming slow because of the lower residence time as it is limiting factor of the degradation process. (c) Mass transfer coefficient is the critical factor in photoreactors because it gives the rate of molecule transfer from bulk to the photocatalyst surface. The mass transfer coefficient was determined using the following equation.

(4) $$V\frac{dc}{dt}=k_{L}A\left(c^{*}-c\right)$$

where V shows solution volume (m^3^), A is coated area (m^2^), c is (mg L^−1^) is a concentration of the pollutant in the bulk of the solution at the time of t (s), and c∗ is a concentration near the solid–liquid interface (mg.L^−1^). By integrating eq (4), $\ln\frac{c^{*}}{c^{*}-c}=k_{L}\frac{A}{V}t$. The mass transfer coefficients showed significant variations as 3.85 × 10^−5^ at 50 mL min^−1^, 8.75 × 10^−6^ at 75 mL min^−1^, 3.73 × 10^−6^ at 100 mL min^−1^ and 1.96 × 10^−6^ at 200 mL min^−1^. This decreasing trend in mass transfer coefficients provides insight into the mass transfer limiting the efficiency of the reactor. A lower flow rate leads to a higher mass transfer coefficient and thus to a higher degradation due to a longer residence time and enhanced interaction between the catalyst and the pollutant. On the contrary, higher flow rates improve mixing and shorten the contact time, resulting in lower mass transfer coefficients and lower degradability.

The analysis of mass transfer and kinetics shows that a high degradation efficiency can be achieved at lower flow rates due to the longer contact time. However, the Peclet number and convective mass transfer increase with the increase of the flow rate, but the shortened residence time leads to a significant decrease in the degradation efficiency of the reactor. Therefore, optimizing the flow rate is very important to achieve a balance between mass transfer and reaction time to achieve maximum degradation efficiency.

### 3.5 Toxicity assessment

The ecotoxicity tests were carried out to assess the effects of the treated and untreated pollutants on the environment using water fleas. The results of the toxicity assessment are shown in Table 9.

**Table 9:** Toxicity assessment findings of the treated solution of studied model pollutants under different parameters.

Pollutant	Flow rate (mL min^−1^)	Number of lights	Toxicity (inhibition percentage)
PB dye	50	3	Non-toxic (0)
PB dye	75	3	Non-toxic (0)
PB dye	100	3	Toxic (100)
PB dye	200	3	Toxic (100)
PB dye	50	6	Non-toxic (0)
IBU	50	3	Non-toxic (0)
IBU	100	3	Toxic (40)
IBU	50	6	Non-toxic (0)
SMX	50	3	Non-toxic (0)
DCF	50	3	Non-toxic (0)

Acute immobilization tests with daphnids were conducted by exposing them to a PB dye solution (concentration = 12 mg L^−1^) prior to degradation. No immobilization of daphnids was observed after 48 h, indicating that the untreated PB dye solution is not toxic to daphnids. Subsequently, toxicity tests of the treated solution obtained from each experiment were performed and found, that the treated solutions of PB dye solution at flow rates of 50 and 75 mL min^−1^ under the illumination of 3 lamps and the treated solution at flow rates of 50 mL min^−1^ under 6 lamps were non-toxic as 100 % of the daphnids were mobilized, whereas the treated solutions at flow rates of 100 and 200 mL min^−1^ under 3 lamps were found to be toxic as 100 % inhibition was observed after 48 h. The efficiency of degradation decreased at higher flow rates. During degradation, transformation products are also formed, which may be more toxic than the parent compound [61,62]. Consequently, the toxicity of the solution was increased.

Toxicity tests were performed with an untreated IBU solution (concentration = 10 mg L^−1^) and 100 % mobile daphnids were visually observed after 48 h. This showed that the IBU solution with a concentration of 10 mg L^−1^ is not toxic as the reported 48hEC_50_ were 51.4 mg L^−1^ [63] and 101.2 mg L^−1^ [64]. The treated IBU solutions with a flow rate of 50 mL min^−1^ at an illumination of 3 and 6 lamps were not toxic to daphnids as 100 % of the daphnids were mobile after 48 h. The treated IBU solution with a flow rate of 100 mL min^−1^ at 3 lamps had immobilized 40 % of the daphnia after 48 h. Similar results were reported in a study that toxic by-products are formed during the degradation process of ibuprofen, which are no longer toxic after complete degradation [65,66].

Prior to the degradation of SMX and DCF, the untreated solutions were also exposed to daphnids for an acute immobilization test. The results showed that both were non-toxic at the concentration used which is consistent with other studies where the reported 48hEC_50_ values were higher than the concentrations tested [64,67,68]. In addition, treated solutions of DCF and SMX at a flow rate of 50 mL min^−1^ with 3 lamps also did not result in any inhibition, as 100 % of daphnids were mobile after 48 h of exposure.

The toxicity evaluation showed that operational parameters such as flow rate can significantly influence not only the degradation of the parent compound but also the toxicity of the treated solution. If the correct operating parameters are not used, the treated wastewater discharged into the environment after treatment can have more harmful effects on aquatic ecosystems than untreated wastewater. Therefore, the use of optimized parameters is necessary to achieve maximum degradation and minimum toxicity.

## 4 Conclusions

This study demonstrated the effectiveness of a flow-through photoreactor with an immobilized photocatalyst TiO_2_ for the degradation of dye Plasmocorinth B and selected pharmaceuticals (ibuprofen, sulfamethoxazole, and diclofenac). Numerical simulations of the flow and radiation field showed that bulk solution is efficiently passed over the glass beads, ensuring the efficient mass transfer, whereas radiation field simulations showed that enough light was reached to the central of the tube that is required to activate the catalyst. The reactor showed high photocatalytic efficiency under optimized conditions, achieving up to 97 % degradation of the dye Plasmocorinth B and complete degradation of ibuprofen and diclofenac. The mineralization rate of organic pollutants was also significantly increased, indicating the complete degradation of the pollutants into non-toxic by-products. The number of lamps and the flow rate were found to have significant effects on photocatalytic degradation. Increasing the number of lamps directly correlated with higher degradation rates, while a higher flow rate led to a lower degradation efficiency. This inverse correlation is attributed to shorter contact time between the pollutants and the photocatalyst. The flow rate has the same effect on TOC removal, while increasing the number of lamps increases TOC removal up to a certain value, after which it starts to decrease. The toxicity tests showed that toxic transformation products were formed at high flow rate, because the removal of the parent compound was low, while at a low flow rate, the efficiency of removal of the parent compound was high and no toxic transformations were formed. This shows that toxic transformation products were formed during the degradation process but were gradually degraded with the complete or 80 % degradation of the parent compound. The continuous flow, high degradation, and TOC removal efficiency of the developed reactor under different operating parameters for a wide range of pollutants provide insight into the implementation of this technology on a large scale. Further optimization of the reactor design, such as the columns consisting of TiO_2_-coated glass beads connected in parallel could improve scalability. Integrating the photoreactor into existing wastewater treatment systems can be an efficient approach to maximizing the removal of contaminants of emerging concern.

## CRediT authorship contribution statement

**Qasim Jamil:** Conceptualization, Investigation, Methodology, Software, Writing – original draft, Writing – review & editing. **Boštjan Žener:** Investigation, Writing – review & editing. **Ula Putar:** Conceptualization, Writing – review & editing. **Lev Matoh:** Conceptualization, Supervision, Writing – review & editing.

## Declaration of competing interest

The authors declare that they have no known competing financial interests or personal relationships that could have appeared to influence the work reported in this paper.
